# Current State-of-the-Art Animal Models of Pediatric Brain Tumors

**DOI:** 10.3390/brainsci15101104

**Published:** 2025-10-14

**Authors:** Tanusri Gudavalli, Fred C. Lam, Santosh Guru, Deyaldeen AbuReesh, Yusuke S. Hori, Susan Hiniker, David J. Park, Steven D. Chang

**Affiliations:** 1 Department of Neurosurgery, Stanford University School of Medicine, Stanford, CA 94305, USA; tgudavalli@dons.usfca.edu (T.G.); fredlam@stanford.edu (F.C.L.); sg928@cam.ac.uk (S.G.); abureesh@stanford.edu (D.A.); yshori@stanford.edu (Y.S.H.); djpark@stanford.edu (D.J.P.); 2 Department of Radiation Oncology, Stanford University School of Medicine, Stanford, CA 94305, USA; shiniker@stanford.edu

**Keywords:** pediatric brain tumors, midline gliomas, medulloblastomas, mouse models, patient-derived xenografts, neuro-oncology, neurosurgery

## Abstract

Brain tumors are unfortunately the most common types of solid tumors in the pediatric population, superseded only by leukemias, and largely bode a poor prognosis. Despite advances in our ability to diagnose and treat pediatric brain tumors, there remains a large unmet need to develop novel therapies to improve patient outcomes. The recent understanding of the molecular drivers of oncogenesis for many of these tumors has led to the engineering of preclinical small animal models which serve as valuable tools for scientists to study the mechanisms of tumor biology, to understand interactions with the tumor microenvironment, and allow for translatable novel therapeutic discovery. This review focuses on the state-of-the art development of preclinical models of two difficult-to-treat pediatric brain tumors: (1) diffuse midline gliomas, the most lethal form of pediatric brain cancer; (2) medulloblastoma, the most common embryonal tumor of the central nervous system. We will then round off this review with a discussion on the emerging use of multi-omics and AI approaches to complement the testing of novel therapies using these in vivo animal models.

## 1. Introduction

Pediatric brain tumors are the leading cause of death in children across all solid tumor types [1], with an estimated annual age-adjusted incidence rate of central nervous system (CNS) tumors of 5.83 cases per 100,000 [2], and an annual age-adjusted mortality rate of CNS tumors of 0.71 cases per 100,000 children between the ages of 0 to 14 years. These statistics establish brain tumors as being the leading cause of cancer death [3]. The 2016 World Health Organization (WHO) reclassification of CNS tumors into molecular subtypes [4], and the more recent 2021 fifth edition WHO CNS5, which builds upon recommendations by the Consortium to Inform Molecular and Practical Approaches to CNS Tumor Taxonomy (cIMPACT-NOW) [5], provide a deeper understanding of the genetic and molecular mechanisms of tumorigenesis for many of these pediatric CNS tumors. This has led to recent advances in developing more representative small animal models of these tumors, allowing for translational research into tumor subtype-specific biology and driving the discovery of precision therapies tailored to each specific tumor subtype [6,7].

**Figure 1:**
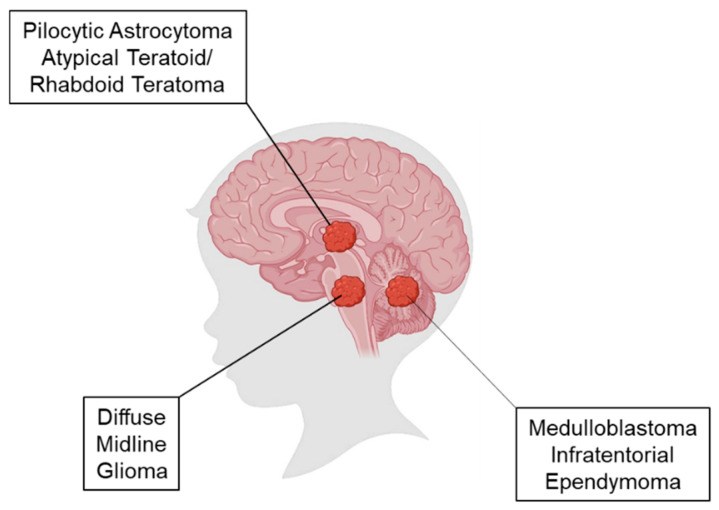
Location of origin of pediatric brain tumors. Representative image of a child’s brain depicts the anatomical location of pilocytic astrocytomas and atypical teratoid/rhabdoid teratoma (ATRT) in the midbrain; diffuse midline gliomas in the pons; and medulloblastoma and infratentorial ependymoma in the cerebellum.

Over the past decade, the development of small animal intracranial models of CNS tumors has served as an invaluable tool for preclinical studies to understand tumor biology and drive novel therapeutic development. These in vivo models can help circumvent the time consuming years and immense numbers of USD required to run equivalent human clinical trials, especially in rare diseases such as primary pediatric CNS tumors [6]. While there are well established and validated patient-derived xenograft (PDX) models of many types of adult primary and secondary brain tumors that fairly faithfully replicate the clinical scenario [8,9,10], PDX models of pediatric brain tumors have only recently come online and many earlier models using immortalized cell lines do not accurately represent the human disease (Figure 1). With the increasing complexity of rare variants of pediatric CNS tumors now being recognized by the WHO CNS5 subcategorization, the ambitions of pediatric oncology research labs can be increasingly challenged to achieve the unmet need to generate these preclinical models. In this article, we review early methods of generating intracranial orthotopic cell line-based xenograft models to the current state-of-the art genome-engineered mouse models (GEMMs) of pediatric brain tumors. We then specifically focus in on how these techniques have been deployed to generate exquisite PDXs and GEMMs of the two most studied and common types of pediatric brain tumors: (1) diffuse midline gliomas (DMGs), the deadliest form of pediatric brain cancer; (2) medulloblastoma (MB), the most common embryonal form of pediatric brain cancer. We have chosen to focus this review on clinical models of DMG and MB and forego focusing on other pediatric brain tumors such as ependymomas and atypical teratoid rhabdoid tumors, which are albeit important topics of discussion, as there has been more research conducted on animal models of DMG and MB than the latter two disease entities.

## 2. Starting Materials Used to Generate Mouse Models of Pediatric Brain Tumors

### 2.1. In Vitro Cell Lines

The basis of understanding pediatric CNS tumor biology relies on in vitro experiments using tumor-derived cell lines to uncover signaling pathways and genome-wide and epigenetic changes that drive tumorigenesis throughout the neuronal development period [11]. There are currently approximately 60 cell lines derived from the majority of pediatric brain cancers [12,13,14]. While the use of human tumor cell lines facilitates the ability to perform scalable multi-omics studies to deepen our understanding of tumor biology and/or perform large library screens for therapeutic drug discovery, they are limited by the lack of inherent tumor heterogeneity that is known to exist in vivo as well as an absence of a representative tumor microenvironment (TME) [12]. Chemogen-induced syngeneic intracranial mouse models of gliosarcomas [15], ependymoblastomas [16], gliomas [17], and oligodendrogliomas that were generated by in utero injections of ethylnitrosourea into the brains of day 15 gestation rat embryos [18], served as valuable immunocompetent preclinical brain tumor models which enabled early studies of the TME and the development of immune therapies [19]. However, yet again, they lack the genetic complexity and temporal spatial heterogeneity of human tumors. Furthermore, these cell lines are grown in a serum-rich environment which alters growth signaling pathways that drive them away from the stem-like nature of CNS tumors, and the selection pressure over multiple passages of these cell lines leads to the creation of a largely singular clonal population [20,21].

### 2.2. Patient-Derived Cell Lines

More recently, patient-derived cell lines (PDCLs) derived from intraoperative tumor samples, grown in serum-free stem cell media, and maintained under low passage, have replaced the use of immortalized human tumor cell lines in generating murine intracranial patient-derived xenografts (PDXs). In particular, these PDCLs exhibit stem-like properties allowing them to be isolated and cultured and maintain self-renewing capability [22]. A subpopulation of CD133^+^ tumor-initiating cells (TICs) was isolated from pediatric gliomas that, when injected into mice, recapitulated features of the primary tumor [23]. This was also seen using cell lines derived from different subtypes of MB, primitive neuroectodermal tumors, and ependymomas [24,25]. When transplanted into the brains of immunocompromised mice, these PDCLs form tumors at a much slower temporal and spatial pace than their immortalized counterparts, allowing them to form intracranial xenografts that are more diffuse and infiltrating, and phenotypically more representative. A biorepository of 18 well characterized and diverse pediatric PDCLs and mouse models is available through the Brain Tumor Resource Lab (BTRL) [26], while the Childhood Cancer Repository also offers a comprehensive biobank of at least 30 well characterized and validated patient-derived orthotopic xenografts (PDOXs) and at least seven cell lines representing 14 molecular subgroups of pediatric brain cancers [6]. This tour-de-force collection of PDOXs and PDCLs, generated through the Children’s Oncology Group ACNS02B3 study, has also undergone in vivo targeted drug sensitivity testing, revealing distinct pharmacogenomic profiles for each distinct molecular tumor subgroup. Alternatively, patient tumor samples can be directly engrafted into immunocompromised mice, bypassing in vitro culture, which allows for the tumor mass to retain its cytoarchitecture, similar to that seen in the nascent brain [27].

### 2.3. Glioma Stem Cell Neurospheres and Cerebral Organoids

As efforts to replicate the in vivo three-dimensional (3-D) structure of glioma stem cells (GSCs) in the ex vivo environment began to evolve, researchers began to grow these GSCs as neurospheres suspended in Matrigel, forming cellular aggregates with hypoxic niches that could be cultured for longer periods of time with radioresistant stem-like properties [28]. The protocols for establishing glioma stem cell neurospheres in suspension have been well established, experimentally validated, and generally involve taking fresh tumor samples from the operating room and immediately dissociating the tissue into single cells within one hour after surgery via enzymatic digestion, maintenance, and passage in serum-free Neurobasal media, fibroblast growth factor, and epidermal growth factor (NBE media) [24,29,30]. GSC neurospheres maintained in this in NBE media retain their tumorigenicity at low passages and maintain the expression of neural stem-like markers including Sox2, nestin, and CD133 [31]. This is in contrast to previous protocols of maintaining neurospheres in media containing 10% fetal bovine serum, which led to a loss of tumorigenicity and significant genetic drift from their original tumor characteristics at late passages [29]. Furthermore, these glioma neurospheres can be cryopreserved, thawed, and maintained in suspension in collagen-coated flasks, and successfully implanted into the brains of immunocompromised mice to generate orthotopic xenograft models of gliomas [30].

More recently, 3D tumor organoid cultures have been developed with the ability to mimic the phenotypic and molecular heterogeneity of different cancer types, including pancreatic [32], prostate [33], liver [34], breast [35], bladder [36], ovarian [37], and gastrointestinal cancers [38]. Tumors of epithelial origin such as the above types can be dissociated and cultured in 5% Matrigel in the presence of exogenous growth factors to form 3D structures. Patient-derived glioblastoma organoids have been generated using a similar protocol with fresh patient tumor tissue samples taken from the operating room and dissociated into single-cell suspensions, with red blood cells removed by brief hypotonic lysis and viable cells sorted by trypan blue [28]. Bao and colleagues described a technique whereby pediatric glioma biopsies were taken from the patient and implanted directly into immunocompromised mice without further processing to develop xenografts [39]. Human xenograft tumors can then be explanted and sorted into a CD133^+^ subpopulation that is enriched in cancer stem cells, expressing neural and/or cancer stem cell markers, including Sox2, Musashi, and Nestin, alongside multilineage differentiation with markers for astrocytes (GFAP, S100b), neurons (Map-2, TUJ1), or oligodendrocytes (O4, GalC). These CD133^+^-enriched glioma stem cells form neurospheres with self-renewal capacity whereas their CD133^−^ counterparts rarely form neurospheres [39]. In another study, Li and colleagues demonstrated that these GSCs exhibited properties of enhanced chemo- and radio-resistance, which led researchers to develop scalable protocols to grow large amounts of 3D GSCs which could be used for in vivo implantation, genome-wide interrogation screening, and small molecule therapeutic screening [40]. Similarly to previous studies, these GSC neurospheres retained stable mRNA levels of glioma stemness markers, including CD133^+^, CD44^+^, CD15^+^, and CD49f^+^ even after 10 passages, maintaining clonal stability, making them ideal tools for a diverse number of downstream applications [40,41]. More recently, 3D glioma organoids have been co-cultured with chimeric antigen receptor T (CAR-T) cells to assess the efficiency of CAR-T antigen specificity for achieving tumor killing [42].

Muguruma and colleagues previously described a technique of combining human epithelial stem cells (hESCs) and inducible pluripotent stem cells (iPSCs) to produce hESC/iPSC aggregates that could be steered to differentiate into cerebellar progenitors and neurons [43]. Studies have shown that using the PiggyBac system to introduce combinations of oncogenes, *Gfi1* + *c-MYC* (GM) or *Otx2* + *c-MYC* (OM), into postnatal cerebellar progenitors can generate Group 3 MB in mice [44,45,46]. Ballabio and colleagues used this combination of GM and OM human cerebellar organoids to generate Group 3 MB intracranial orthotopic xenograft mice and identified SMARCA4 as a druggable target in Group 3 MB [46]. Huang and colleagues showed that the expression of *MYCN* in both otherwise-normal neuroepithelial stem (NES) cells and *PTCH^+/−^* NES cells derived from patients with Gorlin syndrome led to the formation of SHH MB when injected into the cerebellum of mice [47]. Finally, Susanto and colleagues showed that the injection of *PTCH1*-mutated NES cells into the cerebellum of immunocompromised mice generated SHH MB and further identified *LGALS1* as a target gene in SHH MB [48].

## 3. Methods of Generating Mouse Models of Pediatric Brain Tumors

### 3.1. Intracranial Orthotopic Xenograft Mouse Models

**Figure 2:**
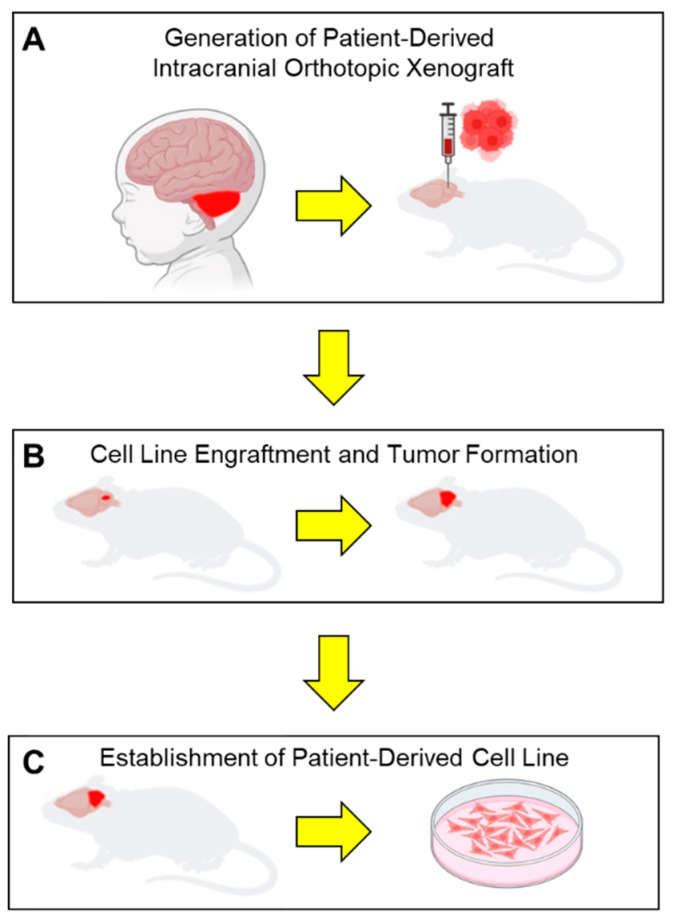
Schematic workflow of establish patient-derived orthotopic intracranial xenografts and orthotopic cell lines. (**A**) Intraoperative brain tumor samples are directly engrafted into the cerebellum of an immunocompromised mouse. (**B**) Mice are monitored for signs of neurologic deficit due to tumor cell engraftment and tumor growth. (**C**) Tumors are explanted from mice after they succumb to their tumor burden and dissociated to generate patient-derived cell lines.

The strain of immunocompromised mice can directly influence the ability to perform downstream applications using these PDXs. The most common strains used include (1) BALB/c mice; (2) severely compromised NOD-SCID mice; (3) Rag1-deficient mice; (4) NCR Nude mice. BALB/c mice are extremely radiosensitive and therefore are an unsuitable strain to use if performing radiation-based studies [49]. NOD-SCID mice have both type 1 and type 2 diabetes and severely abnormal immune systems and therefore are unsuitable for use in immuno-oncological or metabolome-based experiments. NCR Nude mice have innate but not adaptive immune systems, and are able to generate PDXs using immortalized in vitro cells lines, but are generally unable to be used to generate PDXs using TICs due to host rejection. The limitations of all these strains are their obvious lack of intact immune systems, which prevents the development of an essential immune TME. The development of humanized mice, using gamma irradiation to deplete the marrow compartment of NON-SCID mice, followed by a human bone marrow stem cell transplantation, leads to the reconstitution of a compartmentalized human immune system, which can be used to study the interactions between intracranial PDXs and their transplanted human TME. However, humanized mice are expensive to generate, requiring housing in ultra-clean animal facilities, and can experience graft-versus-host disease, and therefore can severely affect the scalability of experiments. The St. Jude’s Children’s Research Hospital has 37 PDX models of pediatric brain tumors that have been thoroughly characterized using whole genome sequencing, DNA methylome profiling, and RNA sequencing [50]. Other limitations of these murine models are that not every PDCL will engraft in the brain, therefore requiring painstaking and time consuming trial and error to find the cell lines that will successfully engraft. Figure 2 depicts the workflow of obtaining an intracranial tumor sample of a pediatric brain tumor and stereotactically injecting these dissociated cells into the brains of immunocompromised mice and waiting for engraftment and then tumor formation.

### 3.2. Genetically Engineered Mouse Models

Genetically engineered mouse models (GEMMs) are the next step up in the state-of-the-art methods of modeling pediatric brain tumors [51]. GEMMs leverage the delivery of gene therapy targeting specific drivers of gliomagenesis, including PTEN, NF1, Ras, EGFR, PDGF, and Akt pathways [52,53], directly into the brains of immunocompetent mice, allowing for the study of tumorigenesis and therapeutic discovery in the syngeneic backdrop of an intact immune system. Danks and colleagues first described the formation of astrocytomas in the brains of transgenic mice expressing the Simian Virus 40 (SV40) T antigen driven by the glial fibrillary acidic protein (GFAP) promoter [54]. Although transgenic mouse models enable immuno-oncology investigations, they are costly to generate and maintain, have a longer latency and higher variability of tumorigenesis, resulting in tumors of mixed histological grading, and may fail to recapitulate key pathological features of gliomas [55,56,57]. Early GEMMs used the tet regulation or Cre-inducible alleles to direct gene expression that was tunable and controllable within specific cellular compartments. Retroviral or adenoviral vectors delivering Cre recombinase (i.e., RCAS/Tva systems) induce the expression of multiple genes in a single mouse using a Tva receptor for subgroup-A avian sarcoma leucosis viruses (ASLVs) [58], while the replication-competent avian leukosis virus splice acceptor (RCAS) viral vectors derived from ASLVs are modified to express multiple oncogenes [59]. The penetrance of the RCAS/Tva virus into cells is low, leading to only a small fraction of cells being infected and a small number of cells being transformed to form a tumor—much like what happens in the development of pediatric brain tumors [60]. More recently, researchers have successfully generated pediatric midline brainstem gliomas using the in utero electroporation of oncogenic plasmids into the embryonic mouse brain [61,62,63]. PiggyBac vectors and Sleeping Beauty transposons have been used with good results in genetically modeling adult and pediatric brain tumors, with tunable properties and quick on/off gene expression, and being deliverable to anatomical locations in the neonatal mouse brain to drive the transformation of progenitor cells [64,65,66].

GEMMs are invaluable for providing insights into the molecular events that drive tumor initiation, progress, and metastasis. They also allow for the modeling and study of the TME [67] and the functions of both the immunosuppressive and pro-tumorigenic immune compartments [20,68,69]. However, since only a limited number of gene alterations are introduced, these mouse models cannot faithfully reflect the overall heterogeneity observed in human gliomas [20]. Furthermore, large-scale therapeutic studies cannot be performed using GEMMs due to high costs incurred with producing and maintaining these mice, as well as the poor control of tumor initiation preventing reproducible results, leaving the majority of drug discovery efforts to be performed using PDCLs [70,71].

A major limitation of both PDXs and GEMMs is the absence of standardized protocols for their establishment and evaluation, which raises challenges for reproducibility across institutions. For PDXs, differences in implantation site, host strain, engraftment criteria, and methods for assessing antitumor activity have historically led to variable outcomes, complicating cross-study comparisons. Recent efforts by the NCI PDXNet Consortium have highlighted this issue and proposed consensus recommendations for harmonizing study design, growth assessment metrics, and reporting standards [72]. Similarly, GEMMs are generated using diverse genetic drivers, promoters, and delivery methods, resulting in variability in tumor latency, penetrance, and histopathology. Without uniform guidelines, the findings obtained in one laboratory may not be directly comparable to those from another, limiting collaborative studies and meta-analyses. The adoption of standardized protocols and shared repositories would substantially improve reproducibility and accelerate the translational impact of these models.

## 4. Mouse Models of H3K27 Mutant and Wild-Type Diffuse Midline Gliomas

Pediatric gliomas are a heterogeneous category of brain tumors, subdivided into low-grade gliomas (LGGs) and high-grade gliomas (HGGs). Of the pediatric HGGs, these are further subcategorized into diffuse midline glioma (DMG) H3-K27-altered, which has distinct molecular and clinical features as well as prognosis. Whole genome next-generation sequencing and DNA methylation profiling have further refined our ability to predict improved survival for patients with histological HGGs that have LGG molecular profiles [73]. Aberrations in growth signaling, proliferation, and angiogenesis (MAPK, EGFR, and VEGF pathways) and pro-oncogenes such as TP53, MYB/MTBL1, BRAF, FGFR, histone H3, and FGFR are commonly seen in pediatric gliomas [74,75,76]. Early GEMMS for pediatric gliomas leveraged *Nf1* and *Trp53* mutations by crossbreeding individual knockout strains to produce the desired *Nf1* and *Trp53* dual-combination knockout, producing tumor-bearing mice that had a range of astrocytomas of all stages with representative histopathological features [53,71]. The generation of *Pten* CNS heterozygotes in these *Nf1/Trp53* double knockout mice led to the formation of high-grade astrocytomas, while *Pten* haploinsufficiency led to the formation of grade 3 astrocytomas, and the *Pten* loss of heterozygosity (LOH) along with Akt activation-accelerated progression into grade 4 astrocytomas [70]. One pitfall of these early GEMMs is that Trp53 alteration is not known to drive gliomagenesis in humans and therefore, the tumors that arise in the brains of the GEMMs may potentially represent metastases as opposed to primary gliomas. The activation of the p21-ras signaling pathway leads to the growth of human astrocytomas with WHO grade-specific features. V12Ha-*Ras* transgenic mice under the control of the GFAP promoter form astrocytomas that have the added genetic complexity of the aberrant expression of p16, p19, and PTEN while overexpressing EGFR, MDM2, and CDK4, with chromosomal rearrangements comparable to human astrocytomas [77,78,79].

High-grade brainstem gliomas, also known as diffuse intrinsic pontine glioma (DIPG) account for ~15–20% of pediatric brain tumors and are one of the leading causes of death among children with brain tumors with a peak incidence between 6 and 8 years. They comprise ~ 50% of all pediatric HGGs with somatic histone H3 mutations as a hallmark distinguishing pediatric vs. adult gliomas [80,81,82]. Histone H3 K27M mutations occur in ~ 80% of DIPGs and other HGGs arising in midline structures such as the thalamus [5]. Given the location of these tumors and the poor penetrance of systemic therapies, the prognosis of HGG patients is dismal [83,84].

Several GEMMs of DIPG have exquisitely recapitulated the human disease phenotype. An earlier GEMM of brainstem glioma by Becher and colleagues in 2010 using the RCAS/tv-a system was derived by injecting 1 μL (10^5^) RCAS-PDGF-B-expressing DF1 cells into nestin tv-a (Ntv-a) or Ntv-a;*Ink4a-ARF^−/−^* mice [85]. As PDGFRα expression is elevated in pediatric high-grade brainstem glioma patients, the researchers infected RCAS-PDGF into the posterior fossa of neonatal Ntv-a mice within 72 h of birth, resulting in the formation of low-grade brainstem gliomas. The tumors were mixed astrocytic and oligodendroglial, but without evidence of embryonal tumors (i.e., medulloblastomas). Becher’s group then went on to further refine their GEMM of DIPG using the RCAS/tv-a system driven by PDGF-B, H3.3K27M, and p53 loss [86].

Larson and colleagues generated conditional knockin mice, *H3f3a^LSL-K27M-Tag/+^,* in which H3.3 K27M is expressed from the endogenous *H3f3a* locus following the Cre recombinase (Cre)-mediated excision of a *loxP*-flanked transcriptional STOP cassette (LSL) [87]. Crossbreeding *H3f3a^LSL-K27M-Tag/+^* mice to *Nestin-Cre* mice which constitutively expressed Cre in neural stem and progenitor cells throughout the CNS generated mice in which mutant H3.3 K27M expression in neural stem cells (NSCs) led to increased NSC proliferation. The further incorporation of an active PDGFRa mutation with p53 loss induced brainstem gliomas in mice resembling that of human DIPG. Gene expression profiling demonstrated the upregulation of genes associated with neural development, hinting at the disruption of neural developmental programs underlying the pathophysiology of DPIGs and pediatric HGGs [88].

More recently, du Chatinier and colleagues developed an immunocompetent DMG mouse by first establishing primary tumor cell lines from murine DMG tumors generated by the intrauterine electroporation (IUE) of PiggyBac DNA plasmids and complementary PiggyBac transposases to introduce H3f3a^K27M^ and Pdgfra mutations and dominant negative p53 into the fourth ventricles of embryonic day 13.5 pups of C57BL/6 immunocompetent mice [71]. This technique of genetically manipulating the midbrains of embryonic pups in utero led to the development of DMG tumors in a temporal and spatial manner mimicking human disease. They were also able to establish neurosphere cell lines from these baby DMG mice and performed pharmacological in vitro validation using panobinostat, a histone deacetylase inhibitor that is currently in phase I clinical trials for children with recurrent disease and has shown promise when combined with an immune checkpoint inhibitor for the treatment of different cancer types. As in utero genetic manipulation techniques become more and more refined over time, it will enable the development of even more accurate modeling of the multiple subtypes of pediatric brain tumors.

Mouse models of the less common H3.3 wild-type (H3.3WT) gliomas have been generated in an RCAS system Nestin-TVA driven by PDGF signaling and p53 loss with p53^flx/flx^ transgenic mice [85,89]. Tumors were induced by injecting 10^5^ virus expressing DF1 cells containing the viral vector RCAS-PDGF-B + RACS-Cre + RCAS-H3.3WT into either the cerebral cortex or brainstem of post-embryonic day 3 neonate mice using a previously published injection protocol [90]. Cortical tumors had a leakier blood–brain barrier (BBB) compared with their brainstem counterparts but this was not dependent upon H3.3K27M status, suggesting that BBB permeability was dependent on the location of tumor induction and not histone mutation status. Furthermore, the mean volume of H3.3WT vs. H3.3K27M tumors did not differ significantly regardless of whether they were induced in the cortex or in the brainstem. In a separate study, du Chatinier and colleagues established three primary H3WT DMG tumor cells lines using their PiggyBac DNA plasmids followed by injection into the fourth ventricles of embryonic day 13.5 pups of C57BL/6 immunocompetent mice [71]. Similarly to their H3.3K27M DMG mouse models, the wild-type DMG models faithfully recapitulated the growth, morphology, and immune microenvironment of human DMG. For the sake of completion, we have included a summary of other published in vivo animal models of pediatric DMG in Table 1.

## 5. Mouse Models of Medulloblastoma

Medulloblastomas (MBs) are a complex and heterogeneous group of tumors that have been molecularly subdivided into four principal subgroups: Wingless (WNT), Sonic Hedgehog (SHH), Group 3, and Group 4. WNT subtypes are predominantly characterized by the *CTNNB1* mutation, account for approximately 10% of all MBs, and have excellent survival. SHH MBs account for about 25% of cases, are characterized by SHH signaling, and are thought to originate from the cerebellum and vermis (Figure 1). Group 3 and 4 MBs account for approximately 60% of all cases but are poorly understood. Given that MBs are the most common embryonal tumors in the CNS, it is heartening to know that they have a collective 5-year survival of over 70%, which bodes a much better prognosis than pediatric HGGs [3].

Most MB PDCLs available for research are derived from *MYC*-amplified Group 3 MBs which grow well in vitro and can withstand multiple passages in culture but display significant genetic drift and acquire new mutations after serial passaging [94]. PDCLs currently exist to address these issues, while researchers have moved towards organoid and other 3D culture models that more faithfully recapitulate the disease for use in PDOX models [46,95,96]. The most utilized method of generating MB PDOXs involves implanting patient-derived tumors directly into the brains of immunocompromised mice and bypassing in vitro passage to avoid culture-related genetic drift [51]. This technique leverages the intracranial stromal environment to maintain the heterogeneous nature of the tumor bulk [97]. Worldwide biobanks have established approximately 15 PDOXs of MB, composed of WNT (*n* = 1), SHH (*n* = 4), Group 3 (*n* = 7), and Group 4 (*n* = 3), with an average engraftment rate of approximately 35% [51,98].

Many GEMMs of the different subtypes of MB exist, with many leveraging a *PCTH1* mutational background to drive the formation of SHH MB [99,100], as well as *MYCN* mutations and *Trp53* loss to generate Group 3 MBs [101,102]. Our earlier mentioned studies of injections of OM human cerebellar organoids in the cerebellum of mice to form Group 3 MBs [46], or injections of NES cells harboring PTCH1 mutations into the cerebellum of mice to form SHH MBs [47,48] are able to faithfully recapitulate the tumor heterogeneity of human MBs in vivo. For a comprehensive review, we refer our readers to the mini symposium topic review by Roussel and Stripay [103]. We also provide for our readers a summary table of available animal models of MB with their advantages and limitations (Table 2).

## 6. Future Technologies Complementing Animal Models of Pediatric Brain Tumors

Emerging technologies such as spatial transcriptomics, single-cell multi-omics, and multifluidic platforms are increasingly being used to complement and validate data gathered using established in vivo models. Although preclinical models are widely used for drug testing, their translational value is best demonstrated through instances of both success and failure. For example, in animal models of DMG, the histone deacetylase inhibitor panobinostat showed potent antiproliferative effects in vitro and demonstrated target engagement in orthotopic PDXs and genetically engineered mouse models; however, systemic administration did not significantly improve survival, mirroring the limited clinical benefit observed in early human trials [110,111]. In medulloblastoma (MB), smoothened pathway inhibition via vismodegib induced tumor regression in preclinical SHH-subgroup models and showed efficacy in early clinical studies, although it later revealed significant and unexpected toxicities—such as growth plate fusion in pediatric patients—that had not been fully predicted by animal models [112,113]. Together, these examples underscore that while preclinical models can faithfully predict certain therapeutic responses, they may also fail to anticipate the toxicity or durability of response, highlighting the need for integrated translational frameworks.

More recently, spatial transcriptomics, single-cell multi-omics, and brain tumor organoid systems have been used for in vitro high-throughput therapeutic screenings that do not incur the high costs associated with animal experiments. An elegant review by Findlay and colleagues highlights the power of these multi-omic tools in dissecting non-genomic and pharmaco-proteogenomic profiles of pediatric DMG tumors that inform treatment response and disease progression [92]. The closing remarks of their article stressed the need to take into consideration the yet-to-be-characterized proteomic heterogeneity of DMG, including the assessment of the posttranslational architecture, to determine the mechanisms that influence immune system avoidance, and the need to integrate genomics-based treatment target identification with pharmacogenomics and pharmacoproteomics analysis into clinical trial readouts to increase the likelihood of achieving long-term survival for patients. Another study by Sturm and colleagues integrated DNA methylation profiling and targeted gene panel sequencing with blinded neuropathological reference diagnostics for a population-based cohort of more than 1200 newly diagnosed pediatric patients with CNS tumors as part of their neuropathology testing panel [73]. The addition of methylation profiling led to the improved detection of therapeutically relevant genetic alterations and cancer predisposition syndromes. A comprehensive review by Nussinov and colleagues suggests that high-resolution single-cell spatial biology imaging performed over the brain’s developmental period could help decipher novel events that uncover key signals that trigger tumor formation over time [114]. A recent study comparing the transcriptomic and proteomic spatial profiling of pediatric and adult H3K27M mutated DMGs showed that H3K27M mutation profoundly impacts tumor cell transcriptomics related to immune and inflammatory signatures, which may provide further insights in CAR-T cell-based therapy research for the treatment of pediatric brain tumors [115]. Finally, a multi-omics analysis of a panel of MB PDOXs revealed that most MBs do not have actionable therapeutic mutations; however, drug screening pointed towards a prior unknown sensitivity of Group 3 PDOXs to actinomycin D, highlighting the need to move towards a functional precision medicine approach rather than a one-size-fits-all approach to treating MB patients [116].

In parallel, microfluidic blood–brain barrier (BBB)-on-chip systems are advancing as physiologically relevant tools for drug screening. These platforms can recapitulate key features of the human BBB, including tight junction integrity and selective permeability, thereby overcoming the limitations of static transwell assays. Notably, Wang et al. demonstrated a pumpless microfluidic BBB model derived from human iPSCs that sustained in vivo-like barrier tightness and produced permeability coefficients comparable to animal studies, supporting its utility for early drug permeability testing [117]. Together, these novel approaches complement animal- and patient-derived models, and their integration may improve the translational predictive power of preclinical studies. As we continue to use more sophisticated multi-omic methods of interrogating tumor samples, we will discover mechanisms driving the pathophysiology of these tumors, with the potential to identify novel therapies to cure these largely fatal pediatric brain tumors [73,88].

## Figures and Tables

**Table 1:** Preclinical models of diffuse midline glioma (DMG).

Study	Model Name	Model Summary	Advantages	Disadvantages
Lin et al., 2019 [91]; Findlay et al., 2022 [92]	Patient-derived cell lines (PDCLs)	Primary tumor cells cultured in vitro from patient biopsies or autopsy samples.	- Useful for large-scale drug screening - Maintain patient-specific mutations - Easy to genetically manipulate	- Low predictive validity: limited in modeling the tumor microenvironment or predicting patient response - Lack native microenvironment - No BBB - Limited tumor heterogeneity
Brabetz et al., 2018 [6]	Patient-derived xenografts (PDX)	Human tumor tissue implanted into immunodeficient mice.	- Moderate to high predictive validity - Preserves tumor architecture and genetic features - Useful for testing therapeutic responses	- Lacks immune system - Limited BBB function - Costly and labor-intensive
Welby et al., 2019 [93]; Findlay et al., 2022 [92]	In utero electroporation (IUE)	Plasmids with H3K27M and co-mutations delivered to the embryonic mouse brainstem.	- Moderate to high predictive validity - Spontaneous tumor formation in native location - Intact immune system - Reproducible and scalable	- Requires technical expertise - May not capture full human tumor heterogeneity
Welby et al., 2019 [93]	Sleeping Beauty transposon system	Random insertion of H2K27M + PDGFRA + shTP53 into mouse neural precursor cells using transposase.	- Moderate predictive validity - Models co-mutation-driven tumor formation - De novo tumor development in brainstem	- Random insertion - Less control over expression levels
Welby et al., 2019 [93]	Syngeneic allograft models	DMG tumor cells from one mouse implanted into genetically similar immunocompetent mice.	- Moderate predictive validity - Immune-competent environment - Compatible with immunotherapy research	- Not fully representative of human tumors - Limited diversity

Abbreviations: PDXs = patient-derived xenografts; BBB = blood–brain barrier; IUE = in utero electroporation.

**Table 2:** Mouse models of medulloblastoma.

Study	Model Name	Model Summary	Advantages	Disadvantages
Brabetz et al., 2018 [6]; Hovestadt et al., 2019 [104]; Sanden et al., 2017 [105]; Shu et al., 2008 [106]; Genovesi et al., 2021 [107]	Patient-derived orthotopic xenografts (PDOX)	Fresh human tissue implanted orthotopically into immunocompromised mice.	- Preserve tumor histology and heterogeneity - Reflects patient-specific genetics - Used for translational drug evaluation	- High cost and low success rate - Lack functional BBB - No immune interactions due to host immunodeficiency
Roussel & Stripay, 2020 [103]	Genetically engineered mouse models (GEMMs)	Mouse models engineered to express or delete tumor relevant genes.	- Mimics natural tumor evolution - Intact immune system and BBB - Enables long-term tracking of tumor progression	- Long latency and technical difficulty - Mouse-specific tumors (not fully humanized) - Limited molecular heterogeneity - Time and resource intensive
Ballabio et al., 2020 [46]; Huang et al., 2019 [47]; Susanto et al., 2020 [48]	Human Cerebellar Organoids	3D cultures of human stem cell-derived brain-like tissue that mimic MB structure and microenvironment.	- Recapitulates human brain architecture - Suitable for drug testing - Models tumor-niche interactions	- No immune or vascular system - Limited scalability
Kawauchi et al., 2017 [102]; Forgev et al., 2018 [108]; Zuckermann etialu, 2015 [109]	IUE	Somatic gene editing via CRISPR in neural precursors in utero or postnatally to drive tumor formation.	- Models human mutations precisely - Rapid model generation - Avoids germline breeding	- Potential off-target effects - Variable efficiency - may not mimic full tumor evolution

Abbreviations: PDOXs = patient-derived orthotopic xenodegularly interspaced short palindromic repeats and CRISPR-associated protein 9; IUE = in utero electroporation.

## Data Availability

Not applicable.
